# Retinol intake and PCOS management: a plasma metabolite and protein analysis via Mendelian randomization and NHANES 2011–2016

**DOI:** 10.3389/fnut.2024.1434648

**Published:** 2024-10-23

**Authors:** Peng Chen, Sha Ni, Qi-Fang Liu, Ling Ou-Yang

**Affiliations:** Department of Obstetrics and Gynecology, Shengjing Hospital of China Medical University, Shenyang, Liaoning, China

**Keywords:** PCOS, Mendelian randomization, drug target, NHANES, retinol intake

## Abstract

**Background:**

Polycystic Ovary Syndrome (PCOS) represents a complex endocrine disorder characterized by a significant interplay with metabolic dysfunction and obesity. This research endeavors to elucidate the causal dynamics among plasma metabolites, proteins, and PCOS, alongside Body Mass Index (BMI), to pinpoint prospective therapeutic interventions.

**Methods:**

This investigation employed Mendelian randomization (MR) analyses combined with data derived from the National Health and Nutrition Examination Survey (NHANES) to explore the relationships between 1,400 plasma metabolites and PCOS, factoring in BMI adjustments. Additionally, the study examined the influence of plasma proteins and performed a retrospective cross-sectional analysis focusing on retinol consumption and testosterone levels.

**Results:**

MR analyses showed metabolite Glycosyl-N-(2-hydroxynervonoyl)-sphingosine (GNS) and protein Keratin 19 (KRT19) were identified as significant markers in the context of PCOS and BMI adjustments. A Phenome-Wide Association Study (PheWAS) underscored the linkage between KRT19 and BMI, while gene-drug interaction findings demonstrated a connection between KRT19 and retinol. Analysis for NHANES data disclosed a negative correlation between retinol intake and testosterone levels, particularly within normal weight and obese cohorts, suggesting the feasibility of dietary interventions for PCOS management.

**Conclusion:**

The study sheds light on the intricate interactions between plasma metabolites, proteins, and PCOS, considering BMI variations, and highlights KRT19 protein as a promising therapeutic target. The outcomes support the integration of retinol consumption into dietary strategies to regulate testosterone levels and potentially alleviate PCOS symptoms, underscoring the necessity for personalized nutritional and therapeutic approaches in the effective management of PCOS.

## Introduction

Polycystic Ovary Syndrome (PCOS) is a complex endocrine pathology in women who are at reproductive age and is related to metabolic disorder (1). The diagnostic criteria for PCOS contains three distinct clinical characteristics—menstrual irregularities, hyperandrogenism (androgen excess), and the detection of polycystic ovaries via ultrasound—espoused by the National Institute of Health, Rotterdam, and Androgen Excess Society (2, 3). Currently, although great progresses have been made in both the acknowledgement of PCOS and the therapeutics (4–7), some limitations including unsatisfactory cure rate, undiscovered mechanism and disorders relapse still remain (8). Hence, it will be helpful to investigate the disease characteristics and therapeutic interventions for understanding and management for patients with PCOS.

Although PCOS is related to metabolic disorder, the interplay between PCOS and metabolic dysfunction is still intricate, manifesting through a myriad of facets. A variety of researches demonstrate that metabolic anomalies, including impaired glucose tolerance, reduced insulin sensitivity, and perturbed lipid metabolism, are intricately associated with PCOS (9–13). Insulin resistance, a major metabolic aberration in PCOS, is significantly correlated with increased metabolic risks such as dysglycemia, dyslipidemia, and hepatic steatosis (13). Approximately 50–70% of women with PCOS are disturbed by insulin resistance (14). Hence, it is necessary to investigate the correlation between metabolite and PCOS. Numerous studies have emphasized the differential metabolite between PCOS and normal people (15–17), but the casual correlations between them are less to be clarified.

Notably, among obese individuals, the PCOS tends to be aggravated. Obesity is defined as an overabundance of body fat and the body fat content is measured by the body mass index (BMI), calculated by the percentage of weight (in kilograms) to height squared (square meters). According to the definition from the World Health Organization (WHO), a BMI of 25–29 kg/m^2^ is named overweight; higher BMI (30 kg/m^2^) is named obesity (18). Some studies found that the prevalence of metabolic syndrome increases in overweight or obese patients with PCOS, highlighting the exacerbating role of excess weight on the metabolic phenotype of the syndrome (19–21). The amplification of metabolic defects in PCOS is further exacerbated by obesity (12, 22). Women with PCOS tend to exert higher obesity rates and a more deleterious metabolic profile, particularly in Hispanic and African American descent (23). An evidence underscores that PCOS may modulate the impact of genetic variants associated with BMI, intimating a genetic interrelation between obesity and PCOS (24). Genome-wide cross-trait analyses have also unveiled loci shared between PCOS and obesity-related traits, underscoring a genetic and causal linkage between the two conditions (25). Furthermore, PCOS has been independently linked to elevated BMI (26). The influence of BMI on the pathophysiology of PCOS contains insulin resistance, hormonal disequilibrium, and reproductive outcomes. Insulin resistance is an intrinsic defect of PCOS and is magnified by a high BMI regardless of PCOS status (14). Additionally, the interrelation between BMI and metabolism as well as endocrine dysregulation in women with PCOS has been documented (27). In essence, the literature substantiates a complex and bidirectional relationship between BMI and PCOS, involving in genetic, metabolic, reproductive, and endocrine domains (24). These evidences demonstrated that BMI as a covariate might unveil novel insights into the etiology of PCOS.

Retinol is a vital vitamin A form, is crucial for cell proliferation, apoptosis, differentiation, and metabolism (28). Numerous studies have demonstrated that Vitamin A can influence the development of obesity and the development of obesity-related diseases, including insulin resistance (29). Retinol-binding protein 4 (RBP4), an important protein in Vitamin A metabolism, have also been emphasized to be involved in the PCOS metabolic disorder (30) and might be a diagnostic markers in PCOS (31). In our analysis, Retinol was also found to be a pivotal drug target in PCOS. Hence, underscoring the correlation of Retinol with PCOS is necessary for personalized nutritional and therapeutic approaches in the effective management of PCOS.

Randomized controlled trials (RCTs) are a gold standard means to test hypotheses at a population level (32, 33). However, RCTs require intensive human resources, cost, and time, and some interventional strategies are not suitable for RCT-dependent assessments. Recently, Mendelian randomization (MR) has gained attention as a complementary method of exploring the casual relationship between the exposure and outcome in diseases, which utilizing the genetic variants with strong associations with exposure as the instrumental variables (IVs). The three main hypotheses of MR are: (1) relevance. Single-nucleotide polymorphisms (SNPs) from genome-wide association studies (GWAS) are used as IVs, and the selected IVs are strongly correlated with exposure; (2) independence. IVs are not associated with other confounding factors; (3) exclusivity. IVs affect outcomes only by exposure (34). This approach might avoid the confounding bias of conventional epidemiological investigations (35). In this study, we conducted a detailed MR analysis alongside a retrospective cross-sectional study to explore the potential causal relationships among plasma metabolites, plasma proteins, and PCOS. Our study highlights KRT19 protein as a viable therapeutic target and supports the integration of retinol consumption into dietary strategies to regulate testosterone levels and potentially alleviate PCOS symptoms. These findings would enrich our understanding to PCOS and enhance therapeutic strategies for patients with PCOS.

## Materials and methods

### Study design

MR is a method to investigate the casual relationship between exposure and outcome. This study was conducted in accordance with the STROBE guidelines (36), ensuring a robust epidemiological investigation. As previously mentioned, the validity of MR hinges on three critical assumptions (37). Hence, in our methodology, we meticulously followed these assumptions. Our analysis employed a two-sample MR approach to examine the casual effects of 1,400 plasma metabolites on PCOS, with or without adjusting for BMI. Subsequently, we reversed the order of association, conducting another two-sample MR to explore the casual effects of PCOS (with adjustment) on the 1,400 plasma metabolites. Additionally, MR was utilized to elucidate the causal relationships between plasma proteins and the positive metabolites identified in the initial analyses. Significant positive plasma proteins were then selected for the construction of a Phenome-Wide Association Study (PheWAS). Drug signature analysis was also conducted focusing on the identified positive plasma proteins.

**Figure 1:**
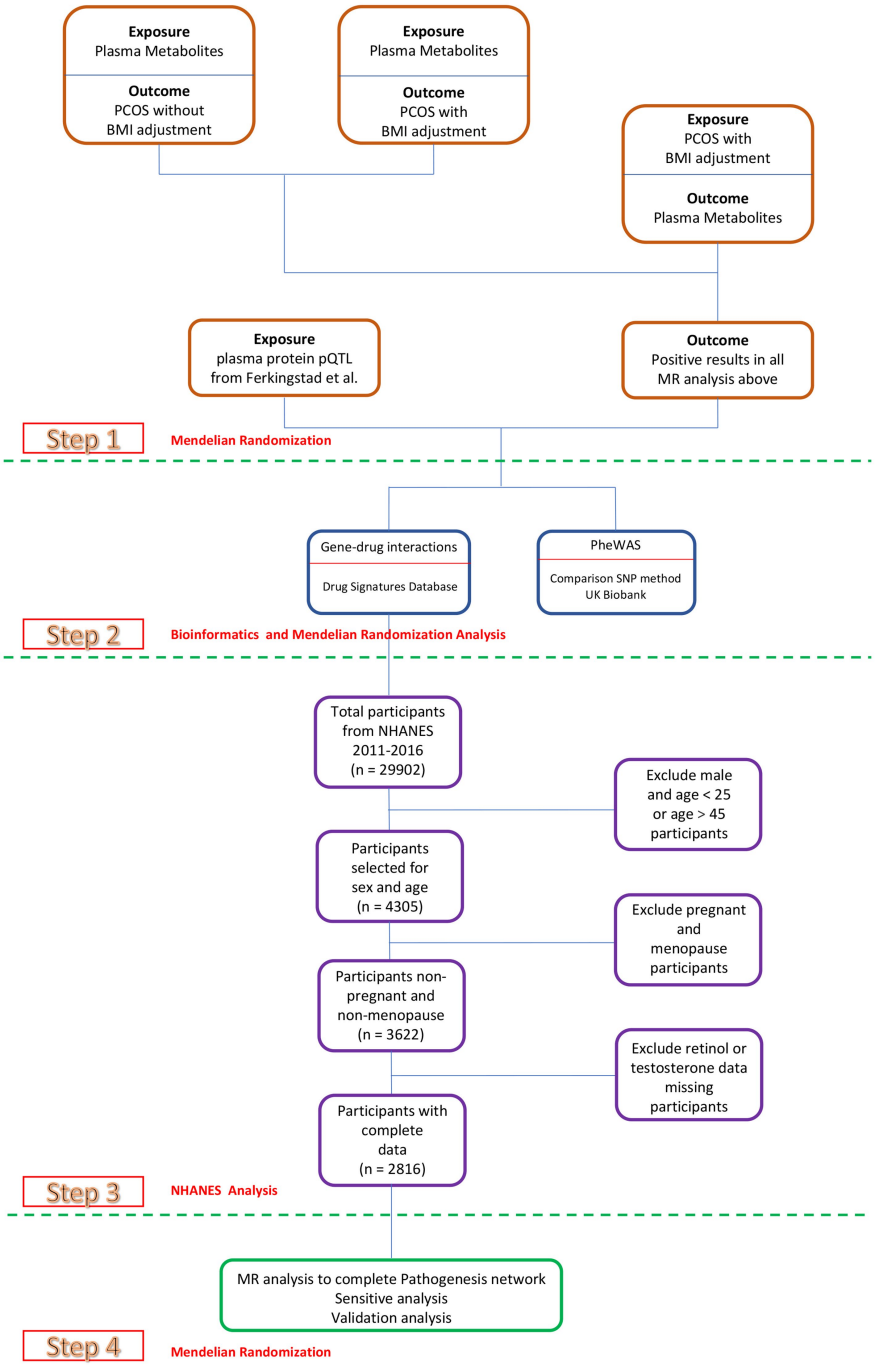
Study design. Overview of the MR study design.

Our comprehensive analysis revealed retinol as the primary outcome of interest. Consequently, we retrieved data from National Health and Nutrition Examination Survey (NHANES), an ongoing study that provides invaluable health-related information on the U.S. adult and child populations, to conduct a retrospective cross-sectional study. This study aimed to investigate the relationships between retinol intake and blood testosterone levels, providing new insights into potential nutritional interventions for PCOS (Figure 1).

### GWAS summary data sources

Data for PCOS were sourced from the investigation conducted by Jaakko et al. (38). The novel genetic variants associated with PCOS within these specific population groups were obtained using data from the FinnGen project and the Estonian Biobank (EstBB). Within the FinnGen cohort, PCOS cases were identified among women documented with the International Classification of Diseases (ICD)-10 code E28.2, ICD-9 code 256.4, or ICD-8 code 256.90. The control group comprised all female participants devoid of a PCOS diagnosis, without imposing any further exclusions. This criterion contained a cohort of 797 cases against 140,558 controls. In the EstBB framework, PCOS identification hinged on the ICD-10 code E28.2, and contained 2,812 cases and 89,230 controls. For the GWAS data adjusted for BMI, a refined association analysis was conducted. It included 482 PCOS cases (60.5% of the original cohort) and 91,631 FinnGen controls (65.2%). Similarly, for the validation dataset, 2,137 PCOS cases (75%) and 68,690 EstBB controls (76.9%) were analyzed with BMI adjustment.

The data concerning 1,400 plasma metabolites were derived from the studies conducted by Chen et al. (39). Additionally, plasma protein pQTL GWAS data were sourced from the research by Ferkingstad et al. (40), while genome-wide association studies (GWAS) data on female bioavailable testosterone levels were obtained from Ruth et al. (41). The analysis also incorporated publicly available GWAS summary datasets for Body Mass Index (BMI) from the MRC-IEU, encapsulating a total sample size of 461,460 individuals. These GWAS summary datasets are meticulously cataloged in Table 1, providing a structured overview of the data underpinning our analysis.

**Table 1:** Description of the contributing GWAS studies.

Traits	PMID	Case size	Sample size	Ancestry
PCOS not adjusted for BMI	34791234	3,609	229,788	European
PCOS adjusted for BMI	34791234	2,619	160,321	European
Plasma Metabolites	36635386	NA	8,299	European
Plasma Protein pQTL	34857953	NA	35,559	European
Female Bioavailable Testosterone	32042192	NA	188,507	European
Body Mass Index	UK Biobank	NA	461,460	European

### SNP selection

We selected exposure-associated independent SNPs with an r^2^ threshold of 0.001 and a distance criterion of ≥10,000 kb, based on their genome-wide (GW) significance levels (*p* < 1.0 × 10^−5^ or *p* < 5.0 × 10^−8^) (42). The F-statistic (F-stat) served as an indicator of instrument strength (IS) and is related to the variance explained by the phenotype. An F-stat value of at least 10 suggests a reduced likelihood of instrument bias (IB) in MR analyses (43). Details regarding the number of SNPs for each causal inference phenotype, along with their respective scale units and F-stats, are documented in Supplementary Table S1.

### Mendelian randomization analysis

First, SNPs were harmonized to the exposure and outcome in an allele-specific manner to ensure proper alignment of effects. Wherever applicable, if any instrumental SNPs for the exposure were absent in the corresponding outcome dataset, a proxy was incorporated using GVs in the linkage disequilibrium (*r*^2^ > 0.8).

We employed an inverse variance-weighted (IVW) meta-analysis approach for MR analysis (44), in addition to the weighted median (45) and MR-Egger regression (MR-ER) approach. Multiple testing was addressed using the false discovery rate (FDR) approach.

### Sensitive analysis for MR

The MR-ER method was further used to assess any potential impacts of the directional pleiotropy (DP) (46) and to perform the MR-pleiotropy residual sum and outlier (MR-PRESSO) method. Data heterogeneity was determined by Cochran’s Q test (47). Leave-one-out (LOO) analysis indicated the influences of a single SNP on overall estimates. To uncover potential reverse causation (RC), genetic instruments were chosen following a strategy akin to that used in MR analysis (48).

“TwoSampleMR v0.5.8,” “MRPRESSO v1.0,” and “MendelianRandomizaiton v0.9.0” packages in R v4.3.1 (source codes: https://github.com/studentyaoshi/MR) were applied for statistical analyses.

### Phenome-wide association studies

To assess the potential implications of key plasma proteins, a Phenome-Wide Association Study (PheWAS) was conducted. A control SNP-set was compiled to benchmark against the plasma protein-associated SNPs for PheWAS analyses, ensuring matches in minor allele frequency (±5%), gene density around SNPs (±50%), proximity to the nearest gene (±50%), and the number of linked SNPs indicating haplotype block size (R^2 ≥ 0.50) (±50%) (49). Data from the UK Biobank was utilized to identify trait associations with the notable plasma proteins. SNP-trait associations that reached nominal significance (*p* < 0.01) were advanced for trait-enrichment analysis (50). The PheWAS explored the relationships between SNPs of significant plasma proteins and over 2000 phenotypic traits, contrasting these findings against the control SNP-set outcomes. Fisher’s exact tests were employed to discern the prevalence of trait associations with significant plasma protein SNPs relative to control SNPs, aiming to identify traits disproportionately associated with plasma proteins risk variants. The FDR correction method was applied to Fisher’s exact *p*-values for trait enrichment to mitigate the risk of false positives due to multiple testing.

### Gene-drug interactions

Drug Signatures Database (DSigDB) was used to identify drug molecules that interact with plasma proteins through the Enrichr platform.^1^

### NHANES analysis

The NHANES study collects comprehensive data through home interviews and health screenings conducted at mobile examination centers. The interviews cover areas such as demographics, socioeconomics, diet, health. And the examination includes detailed medical, dental, physiological assessments, and laboratory tests by professional staff. Dietary data, focusing on intake over the past 24 h, are derived from a two-day recall survey to analyze food and beverage consumption and their nutritional values. Specifically, the study examines total two-day retinol intake and blood testosterone levels from 2011 to 2016, following the ethical standards set by the 1964 Declaration of Helsinki. The study protocol received approval from the National Center for Health Statistics Research Ethics Review Committee, ensuring all participants provided informed consent.

In alignment with the guidelines stipulated by the Centers for Disease Control and Prevention (CDC), our study employed weighted sampling methodologies, complemented by stratification and clustering techniques, to ensure a representative reflection of the United States population. The demographic attributes of study participants were showed as mean ± standard deviation (SD) for continuous variables and percentages for categorical variables. To explore the associations between retinol intake and testosterone levels, we applied multivariable linear regression models. Model 1 was devoid of any covariates. Model 2 incorporated adjustments for age, race, educational attainment, and marital status. Model 3 expanded upon Model 2 by further adjusting for hypertension, diabetes, abdominal diameter, smoking status, and alcohol consumption. Moreover, we conducted a subgroup analysis, stratified by BMI categories: normal weight (BMI < 25), overweight (25 ≤ BMI < 30), and obesity (BMI ≥ 30), employing multivariable regression techniques. To address potential non-linear relationships between retinol intake and testosterone levels, we utilized smoothing techniques and generalized additive models. Statistical analyses were performed utilizing R v4.3.1^2^ and EmpowerStats v4.0^3^, with a predetermined significance threshold set at *p* < 0.05 for denoting statistical significance.

### Data availability

All relevant data for this study are included in the article or uploaded as Supplementary material. Full GWAS summary statistics for the exposure and outcome data used in the manuscript can be found at https://www.ebi.ac.uk/gwas, https://gwas.mrcieu.ac.uk/.

## Results

### Step 1. Metabolic related druggable targets for BMI adjusting PCOS

**Figure 2:**
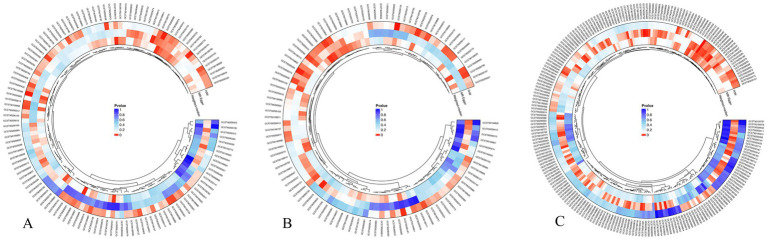
MR analysis between plasma metabolites and PCOS. **(A)** The casual association between plasma metabolites and PCOS without BMI adjustment. **(B)** The causal association between plasma metabolites and PCOS with BMI adjustment. **(C)** The casual association between PCOS with BMI adjustment and plasma metabolites.

In this investigation, we aimed to explore the causal correlations between plasma metabolites and PCOS, with a particular emphasis on adjustments for BMI. In our preliminary analysis, MR analysis was conducted to assess the casual effects of approximately 1,400 plasma metabolites on PCOS under two distinct conditions: with and without BMI adjustment. After adjusting for the FDR, no outcomes of IVW reached statistical significance were discovered. Hence, a threshold of *p*-value less than 0.05 was adopted to denote statistical significance. PCOS without BMI adjustment revealed 77 significantly associated plasma metabolites, comprising of 36 beneficial and 41 deleterious metabolites (Figure 2A). Within the context of PCOS adjusted for BMI, we identified 62 plasma metabolites with significant correlations—divided evenly into 31 metabolites with beneficial and 31 with deleterious effects (Figure 2B). Remarkably, 20 metabolites were causally correlated with PCOS regardless of BMI adjustment, including 12 beneficial and 8 deleterious metabolites.

**Figure 3:**
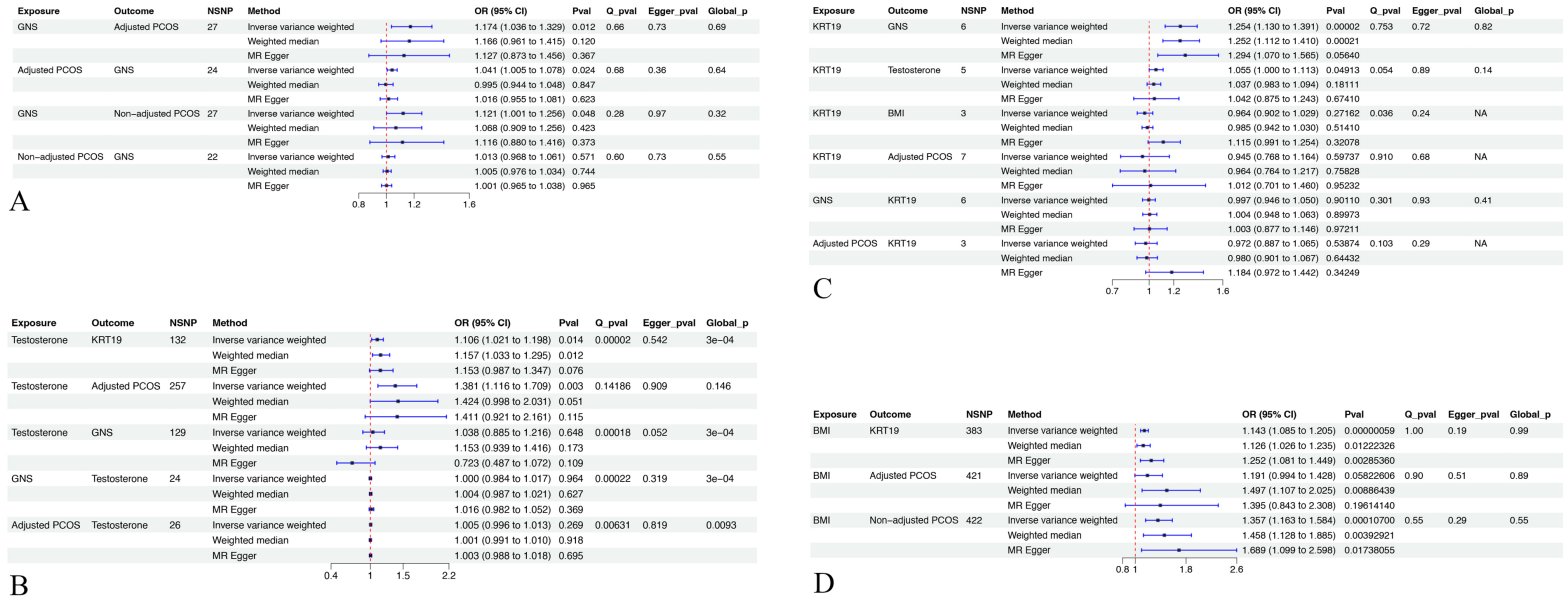
MR analysis and sensitive analysis of related exposures and outcomes. **(A)** MR and sensitive analysis related to GNS. **(B)** MR and sensitive analysis related to Testosterone. **(C)** MR and sensitive analysis related to KRT19. **(D)** MR and sensitive analysis related to BMI. GNS, Glycosyl-N-(2-hydroxynervonoyl)-sphingosine (d18:1/24:1(2OH)). KRT19, Keratin 19. NSNP, number of single-nucleotide polymorphism. Q_pval, *p* value of Cochran’s Q test. Egger_pval, *p* value of MR-ER method. Global_p, *p* value of MR-PRESSO.

Subsequently, we explored the effects of PCOS with BMI adjustment on the same set of 1,400 plasma metabolites. T A total of 64 metabolites were demonstrated to be significantly affected by PCOS with BMI adjustment, featuring 23 with beneficial and 41 with deleterious effects (Figure 2C). When intersected with the 20 metabolites identified in the initial phase, only the levels of Glycosyl-N-(2-hydroxynervonoyl)-sphingosine (d18:1/24:1(2OH)) (GNS) were consistently observed in both sets of positive results, exerting an effect on PCOS (95% Confidence Interval [CI]: 1.0363 to 1.3294, *p* = 0.0117) and being influenced by PCOS (95% CI, 1.0053 to 1.0779, *p* = 0.0241) (Figure 3A; Supplementary Tables S1, S2).

Expanding our scope to the effects of plasma proteins in conjunction with GNS, and after FDR adjustment, Keratin 19 (KRT19) was found to exhibit a significant positive association (95% CI: 1.1301 to 1.3912, pFDR = 0.0251), whereas BIRC2 (95% CI: 0.836 to 0.9248, pFDR = 0.0022) and ING4 (95% CI, 0.8748 to 0.9506, pFDR = 0.0251) displayed significant negative relationships (Figure 3C; Supplementary Table S2). Based on these findings, KRT19 emerges as a viable druggable target for the management of PCOS, particularly when adjustments for BMI were considered.

### Step 2. PheWAS and gene-drug interactions for KRT19

The results from the PheWAS can be interpreted to delineate the correlations between the genetic determination of protein expression and the incidence of specific diseases or traits. Initially, we identified that six SNPs were associated with KRT19 through prior MR analyses. These were subsequently subjected to clumping, with each being matched to four control SNPs (Supplementary Table S3). Following this, PheWAS analyses were conducted utilizing the UK Biobank database to examine each KRT19-associated variant and its control counterparts for associations with 2,502 traits. After adjustment for FDR, only BMI was demonstrated to be a statistically significant with KRT19 SNPs over control SNPs, achieving a significance level of pFDR<0.2 (pFDR = 0.0561) (Figure 3D; Supplementary Table S4). Further MR analysis revealed that BMI exhibited a positive effect on KRT19 expression (95% Confidence Interval (CI): 1.0847 to 1.2048, *p* = 5.8506e-7) (Figure 3D; Supplementary Table S4).

**Figure 4:**
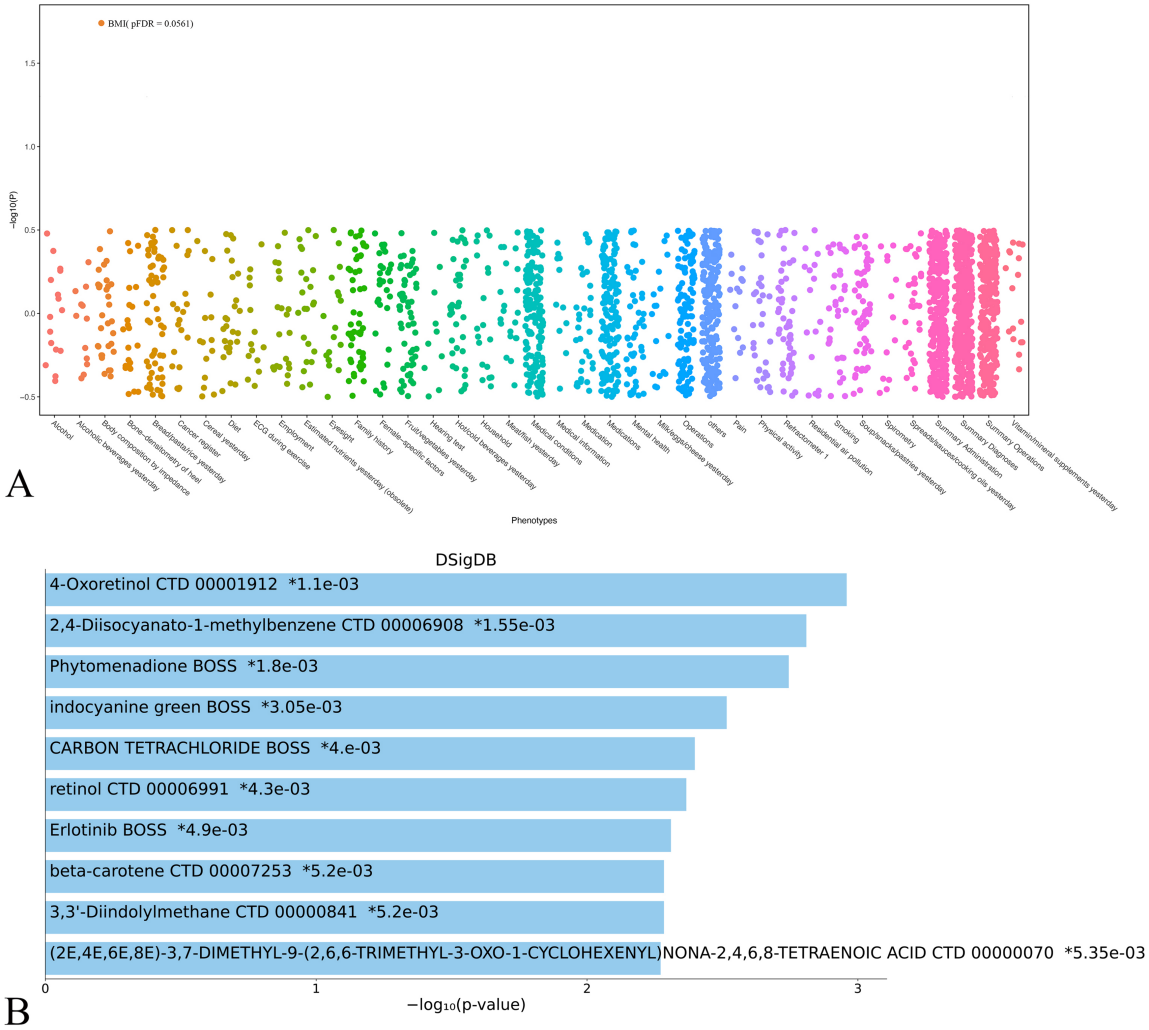
PheWAS and gene-drug interactions for KRT19. **(A)** Manhattan plot of the PheWAS analysis results for KRT19 in UK Biobank database. **(B)** Bar chart of top enriched terms of KRT19 from the DSigDB gene set library. The top 10 enriched terms for the input gene set are displayed based on the −log10(*p*-value), with the actual *p*-value shown next to each term. The term at the top has the most significant overlap with the input query gene set.

Subsequently, based on the DSigDB database, Enrichr analysis was conducted to explore the interplay between KRT19 and potential drug candidates (Figure 4). The results highlighted the significance of Vitamin A metabolites—specifically 4-Oxoretinol, retinol, and beta-carotene—among the top 10 drug candidates for KRT19, meriting further attention (Figure 4B).

### Step 3. NHANES analysis between retinol and blood testosterone

Data from 29,902 individuals registered in NHANES database spanning from 2011 to 2016 were initially considered. Following a meticulous final screening process, a cohort of 2,816 individuals who satisfied the specified inclusion and exclusion criteria were selected for enrollment (Figure 1). These individuals were subsequently categorized into three groups based on their BMI, with the characteristics of each group detailed in Table 2.

**Table 2:** Weighted characteristics of study samples based on BMI.

	Normal weight	Overweight	Obesity	*p* value
	BMI <25 (*n* = 1,017)	BMI > =25, <30 (*n* = 715)	BMI > =30 (*n* = 1,084)	
**Age**	31.464 ± 7.797	32.406 ± 7.250	32.921 ± 7.312	<0.001
**BMI**	21.825 ± 1.986	27.325 ± 1.464	37.223 ± 6.326	<0.001
**Abdominal Diameter**	17.040 ± 1.578	20.284 ± 1.722	25.844 ± 3.479	<0.001
**Retinol Intake (mcg)**	364.854 ± 268.146	357.853 ± 246.628	368.491 ± 289.725	0.72
**Testosterone Level (ng/dL)**	26.270 ± 16.163	28.275 ± 27.345	27.668 ± 15.782	0.08
**Race**				<0.001
Mexican American	92 (6.999%)	156 (15.877%)	215 (15.151%)	
Other Hispanic	108 (7.282%)	82 (9.19%)	109 (7.879%)	
Non-Hispanic White	385 (63.733%)	227 (54.926%)	336 (51.623%)	
Non-Hispanic Black	125 (7.043%)	150 (12.273%)	341 (20.365%)	
Other Race	307 (14.943%)	100 (7.735%)	83 (4.982%)	
**Education**				<0.001
Below High Scholl	105 (8.417%)	138 (13.905%)	202 (14.725%)	
High School	141 (13.604%)	119 (15.151%)	236 (20.717%)	
Above High School	771 (77.979%)	458 (70.944%)	646 (64.558%)	
**Marital Status**				0.05
Yes	588 (60.55%)	433 (64.262%)	604 (58.354%)	
No	429 (39.45%)	282 (35.738%)	480 (41.646%)	
**Alcohol Drink**				<0.001
Yes	635 (78.783%)	414 (71.297%)	662 (71.939%)	
No	272 (21.217%)	219 (28.703%)	313 (28.061%)	
**Smoke**				0.02
Yes	260 (30.73%)	180 (28.783%)	359 (34.932%)	
No	757 (69.27%)	535 (71.217%)	725 (65.068%)	
**Hypertension**				<0.001
Yes	65 (5.979%)	78 (11.152%)	289 (23.389%)	
No	952 (94.021%)	637 (88.848%)	795 (76.611%)	
**Diabetes**				<0.001
Yes	4 (0.225%)	27 (2.581%)	88 (7.4%)	
No	1,013 (99.775%)	688 (97.419%)	996 (92.6%)	

Mean ± SD for continuous variables, *p*-values were obtained using the weighted linear regression model and Kruskal-Wallis rank sum test. % for categorical variables and *p* value was calculated by weighted chi-square test.

In the comprehensive analysis conducted under Model 3—which accounted for confounding factors such as age, race, educational attainment, marital status, hypertension, diabetes, abdominal diameter, smoking status, and alcohol consumption—a significant negative correlation between retinol intake and testosterone levels was identified (Table 3).

**Table 3:** The association between retinol intake (mcg) and testosterone (ng/dL).

Model	Total	BMI = <25	BMI > =25, <30	BMI > =30
	β (95% CI) *p* value	β (95% CI) *p* value	β (95% CI) *p* value	β (95% CI) *p* value
Model 1	−0.004 (−0.007, −0.002) 0.00178	−0.004 (−0.008, −0.000) 0.04277	−0.002 (−0.010, 0.006) 0.62966	−0.006 (−0.009, −0.002) 0.00053
Model 2	−0.004 (−0.007, −0.002) 0.00194	−0.004 (−0.008, −0.000) 0.03658	−0.001 (−0.010, 0.007) 0.73320	−0.006 (−0.009, −0.003) 0.00024
Model 3	−0.004 (−0.007, −0.001) 0.00473	−0.005 (−0.009, −0.000) 0.02933	−0.000 (−0.010, 0.010) 0.99756	−0.006 (−0.009, −0.003) 0.00059

No covariate was adjusted in Model 1. Model 2 indicates that analysis was adjusted for age, race, educational attainment, and marital status. Model 3 indicates Model 2 adjustment plus the adjustment for hypertension, diabetes, abdominal diameter, smoking status, and alcohol consumption.

Subgroup analyses revealed that among the normal weight (BMI ≤ 25) and obesity groups (BMI ≥ 30), significant negative correlations between retinol intake and testosterone levels were observed (Table 3). Conversely, in the overweight group (BMI ≥ 25, < 30), no significant correlation was detected.

**Figure 5:**
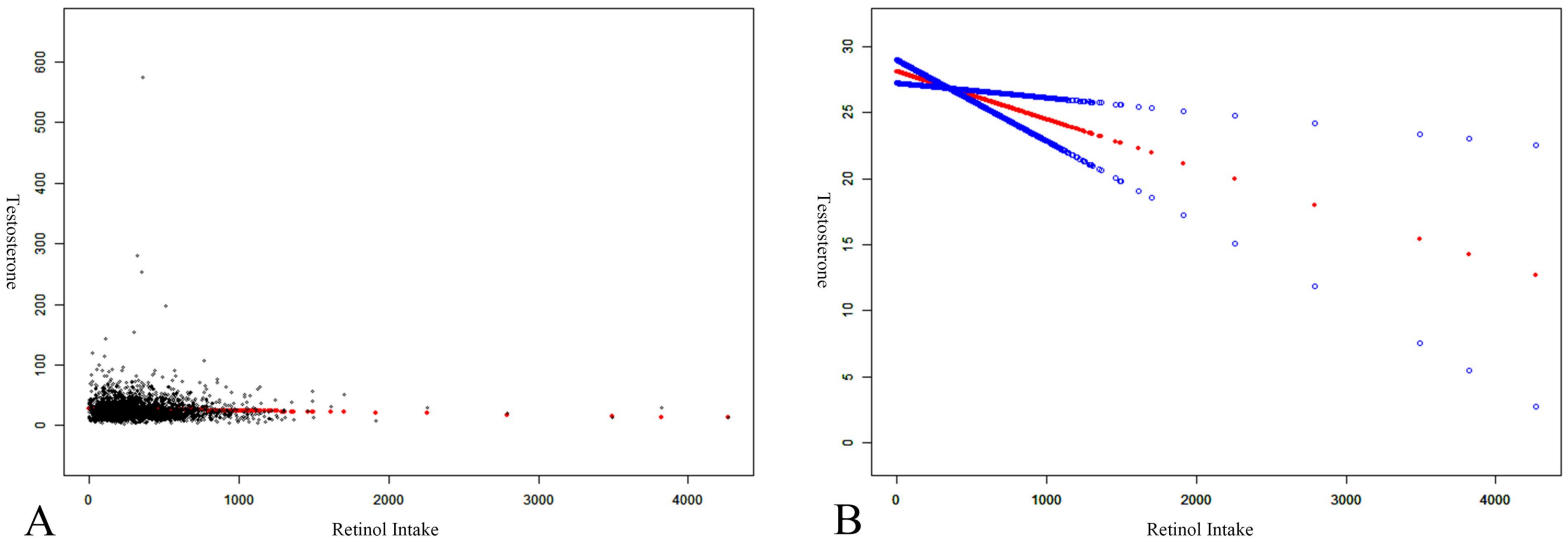
The association between retinol intake (mcg) and testosterone (ng/dL). **(A)** Each black point represents a sample. **(B)** Solid red line represents the smooth curve fit between variables. Blue bands represent the 95% confidence bands derived from the fit.

Further examination was conducted to elucidate non-linear relationships between retinol intake and testosterone levels through smooth curve fittings and generalized additive models, illustrated in Figure 5. The results indicated that retinol intake was negatively correlated with testosterone levels.

### Step 4. Pathogenesis network of BMI adjusting PCOS

Upon synthesizing the results obtained, we developed a conceptual framework illustrating the influence of BMI and retinol on the PCOS after adjusting for BMI. However, certain interactions between mediators remained unanalyzed. To clarify these interactions, MR analysis was employed, the results of which were depicted in Figure 3B. The analysis revealed that testosterone displayed a significant positive association with both BMI-adjusted PCOS (95% Confidence Interval (CI): 1.1158 to 1.7089, *p* = 0.003) and Keratin 19 (KRT19) (95% CI: 1.0273 to 1.1716, *p* = 0.0057). Concurrently, KRT19 exhibited a significant positive association with testosterone (95% CI: 1.000 to 1.113, *p* = 0.0491) (Figure 3C). However, no association was found between BMI-adjusted PCOS and testosterone (Figure 3B, *p* = 0.269).

To validate the association between BMI and PCOS, we investigate the correlations between BMI and PCOS data with or without BMI adjusting. Positive associations were observed exclusively in the cases where BMI was adjusted. Further investigations were conducted to explore the direct associations between testosterone and GNS, KRT19 and BMI-adjusted PCOS, non- adjusted PCOS and GNS, yet no positive findings emerged. Additional reverse causality analyses and sensitivity analyses pertinent to all MR study were also performed, with results detailed in Supplementary Table S5. The results indicated that no heterogeneity and no horizontal pleiotropy were observed between the genetic IVs, indicating the robust of the MR analysis.

**Figure 6:**
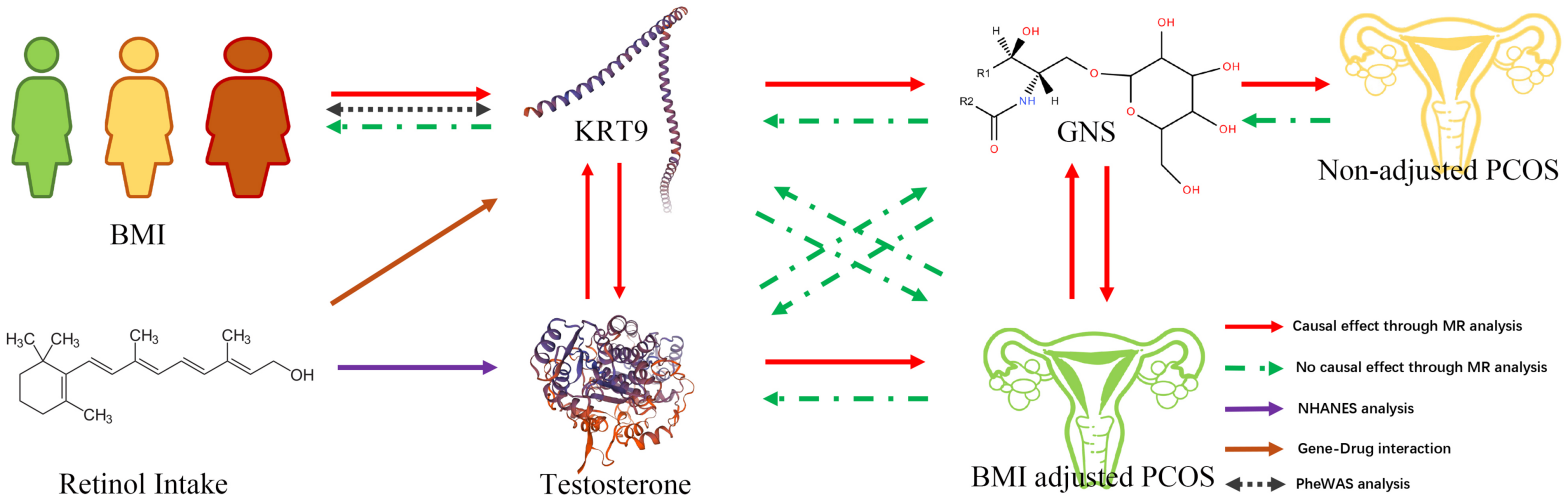
Pathogenesis network of BMI adjusting PCOS. Pathogenesis network overview of BMI adjusting PCOS.

Based on the comprehensive analysis of the data collected, we were able to delineate a clear pathogenetic network for BMI-adjusted PCOS, as illustrated in Figure 6. This enhanced understanding underscores the complex interplay between BMI, retinol, genetic factors, and plasma metabolites in the development of PCOS, particularly when adjusted for BMI.

## Discussion

PCOS is recognized as a prevalent endocrine disorder affecting women of reproductive age. The prevalence of obesity in women diagnosed with PCOS is notable, intensifying the syndrome’s clinical symptoms and adding to its complexity (51). Therefore, the exclusion of BMI as a confounding factor plays a crucial role in uncovering the underlying causes of PCOS. In this study, we employed MR analysis alongside analyses of data from NHANES to explore plasma metabolites and druggable targets that were associated with PCOS after adjusting for BMI. Through MR and gene-drug interactions analysis, KRT19 emerged as a potential therapeutic target, with its effects mediated by GNS and influenced by both BMI and retinol intake. Subsequent multivariable linear regression analysis on individuals from NHANES demonstrated that retinol intake could decrease testosterone levels in individuals of normal weight and those classified as obese, but it did not have a significant effect on the overweight group. These findings suggested that retinol intake and BMI may influence the development of PCOS through mechanisms mediated by KRT19 and GNS.

Glycosyl-N-(2-hydroxynervonoyl)-sphingosine (GNS) plays a pivotal role in cell growth, proliferation, apoptosis, and metabolic regulation (52–54). The critical involvement of GNS in cellular survival and metabolic stability, particularly within adipocytes, and its interconnection with cholesterol regulation (55), highlight its significance. Decreases in plasma sphingomyelins, which may be related to GNS, could reflect changes in lipoprotein cholesterol or deficiencies in sphingomyelin synthesis (56). GNS has also been linked to diabetic polyneuropathy in type 2 diabetes, emphasizing its importance in metabolic and neurological disorders (56). Further research by Guan et al. (57) indicates an inverse relationship between GNS levels and physical activity. In a Puerto Rican study (58) assessing metabolites and cognitive function in a non-diabetic population, lower GNS levels are associated with better cognitive performance, suggesting its potential impact on cognitive health. These insights collectively underscore the critical role of GNS in health and disease, warranting more in-depth exploration. In our investigation, findings indicated a bidirectional relationship between GNS and PCOS. Specifically, GNS could contribute to the exacerbation of PCOS, while simultaneously, the presence of PCOS might also lead to an increase in GNS levels. This reciprocal interaction underscores the complexity of the metabolic interplays underlying PCOS, highlighting GNS’s significant role in this dynamic.

KRT19, a constituent of the keratin family, plays a significant role in numerous processes associated with cancer (59–62). In our investigation, KRT19 functioned as a central node within the pathogenesis network for PCOS modulated by BMI. It has been observed that KRT19 can enhance the progression of PCOS via GNS and is positively influenced by BMI. Furthermore, KRT19 shares a bidirectional relationship with testosterone levels and is subject to modulation through retinol consumption. The molecular mechanisms of KRT19 include several pathways, such as the MET-ERK1/2-AP1 and SP1 axis (63), the potentiation of Notch1 signaling by Linc-KILH (64), DNA methylation-associated allelic inactivation (65), and immune infiltration (66). However, these findings do not definitively elucidate the results we obtained. A more detailed investigation into the role of KRT19 in PCOS is warranted for a comprehensive understanding.

Nutritional supplements and complementary therapies have been applicated in the treatment of PCOS (7, 67, 68). Retinol, a vital vitamin A form, is crucial for cell proliferation, apoptosis, differentiation, and metabolism (28). It is obtained from dietary beta-carotene and retinyl esters. In the gut, retinol converts to retinyl esters, then to chylomicrons for liver transport. There, it is stored or becomes retinol and its derivatives (69). Mammalian cells can absorb retinol via STRA6, metabolizing it into retinal and retinoic acid forms like 4-oxo-retinoic acid. Our research identified 4-oxoretinol, retinol, and beta-carotene as top KRT19 drug candidates, with beta-carotene converting to retinol, then to 4-oxoretinol. Thus, we focused on retinol for our study, emphasizing its therapeutic potential against KRT19. Retinol has also been demonstrated to be related to insulin resistance (29). In PCOS, RBP4, an important protein in Vitamin A metabolism, has also been emphasized to be involved in the PCOS metabolic disorder (30, 31). Our findings indicated that for women aged 20–45, consuming retinol can decrease testosterone levels in individuals with a BMI below 25 and above 30, with no significant impact observed within the 25 ≤ BMI < 30 range. In women with a normal BMI, a deficiency in retinol intake may increase testosterone levels and GNS through KRT19, potentially leading to the development of PCOS, which can further result in weight gain. However, for those who are overweight, retinol intake does not effectively modify testosterone levels, may lead to a situation where GNS and non-adjusted PCOS, present a one-way relationship. This pattern suggests that retinol intake could play a role in the prevention of PCOS among women with a normal BMI, yet its capacity to serve as an effective treatment in overweight individuals is limited.

## Limitations

This research is subject to several constraints. Initially, our analysis focused on the pathogenic effects of proteins reported in various studies, where discrepancies in measurement techniques across these studies could have introduced biases into our findings. Additionally, while the MR and NHANES study populations and datasets encompass diverse racial and ethnic groups, the inherent inconsistencies among these groups might have led to biases in the results. Moreover, the casual correlation between GNS and PCOS, as well as the targets KRT19 in PCOS are not fully confirmed in the present analysis. More experiment validation and clinical validation are necessary to performed to improve this aspect of knowledge.

## Conclusion

Employing a variety of methodologies, including Mendelian randomization, gene-drug interaction analyses, and retrospective cross-sectional studies, our investigation reveals that circulating KRT19 could potentially induce BMI-adjusted PCOS via mechanisms involving GNS and testosterone. Both BMI and retinol consumption appear to modulate KRT19, influencing its impact on PCOS. Notably, retinol intake might contribute to PCOS prevention in women with a normal BMI by affecting KRT19 activity. Furthermore, KRT19 emerges as a promising metabolic-related, druggable target for PCOS management and treatment.

## Data Availability

The original contributions presented in the study are included in the article/Supplementary material, further inquiries can be directed to the corresponding author.

## Footnotes

^1^ https://maayanlab.cloud/Enrichr/

^2^ http://www.Rproject.org

^3^ http://www.empowerstats.com
